# Base editing reversal of radiation sensitivity in *NHEJ1* immunodeficiency

**DOI:** 10.70962/jhi.20260068

**Published:** 2026-09-03

**Authors:** Renuka Kadirkamanathan, Roland Preece, Lisa Woodbine, Elena Korneeva, Arina Lazareva, Winnie Ip, Waseem Qasim

**Affiliations:** 1 Molecular and Cellular Immunology, Institute of Child Health, Zayed Centre for Research, London, UK; 2 Genome Damage and Stability Centre, University of Sussex, Brighton, UK; 3 Great Ormond Street Hospital for Children NHS Trust, London, UK

## Abstract

Inherited defects of DNA double-stranded break (DSB) repair can result in radiosensitive/radiation-sensitive (RS) SCID (RS-SCID). We applied base editing to reverse *NHEJ1* mutations in patient fibroblasts, exemplifying how this technology can help interrogate RS sequence variants.

## Teaser

The cutting and joining of DNA is a key step in immune cell development, but inherited errors in this process can cause individuals to be born without functioning immune systems. We can use molecular pencils, called base editors, to modify DNA sequences in order to study newly identified mutations, or those of unknown significance, that may be responsible for these conditions.

## Introduction

Inherited defects of DNA double-stranded break (DSB) repair can impede critical pathways required for the generation of diverse T and B cell antigen-specific receptors and are often caused by mutations in genes encoding nonhomologous end joining (NHEJ) machinery, including Artemis (*DCLRE1C*), ligase IV (*LIG4*), and XRCC4-like factor XLF (*NHEJ1*). This can give arise to a spectrum of clinical presentations, ranging from mild growth failure or neurodevelopmental delay to “radiosensitive”/“radiation-sensitive**”** (RS) severe combined immunodeficiency (RS-SCID), similar to T^-^B^-^ SCID disorders typical of mutations in *RAG 1* and/or *RAG2*. Historically, diagnostic investigations for RS disorders involved the detection of pathogenic mutations in DSB repair pathway genes alongside *in vitro* assessments of fibroblast sensitivity to ionizing radiation and resulting DSB repair capacity (1). These are usually triggered after a suspicion of SCID has arisen or immunological studies have revealed abnormal T or B cell numbers or skewed receptor repertoires. Newborn screening for SCID, which can identify reduced or absent T cell receptor excision circles (TRECs), and wider genetic assessments for growth or neurodevelopmental delay, have led to more frequent investigations. As a result, sequence variants with both anticipated and uncertain significance are being increasingly reported. An ability to reverse mutations and undertake subsequent functional studies in primary patient cells would assist disease modelling and clinical diagnostics as well as subsequent management. An evolving CRISPR toolbox now offers numerous avenues to introduce or reverse genetic changes in a highly targetable manner. Conventional “knock in” strategies employ DNA templates to insert sequences through precise Cas9 nuclease–mediated DSBs and homology-directed repair (HDR). Alternatively, base editing can deliver efficient C>T (cytidine base editor, CBE) or A>G (adenine base editor, ABE) conversions without DSB formation by combining CRISPR guidance machinery with catalytically programmed nickases and cytidine or adenine deaminase modules, respectively (2). We applied adenine base editing to reverse sequence variants in *NHEJ1*, which had been detected in two unrelated RS-SCID patients (P1 and P2). Our approach used pre-established stocks of mRNA encoding different base editors, which had previously been validated in healthy donor as well as patient fibroblasts using electroporation and single gRNA (sgRNA) targeting control sites such as *B2M*, the conserved domain of HLA-class I. Bespoke sgRNAs for *NHEJ1* variant reversal were then commercially procured and assessed for on- and off-target activity in patient fibroblasts. Restoration of protein expression and resolution of DSB repair were achieved, establishing a pathway for rapid investigation of such RS sequence variants.

## P1: *NHEJ1* c.532C>T p.R178* reversal with SpCas9-ABE

**Figure 1.**
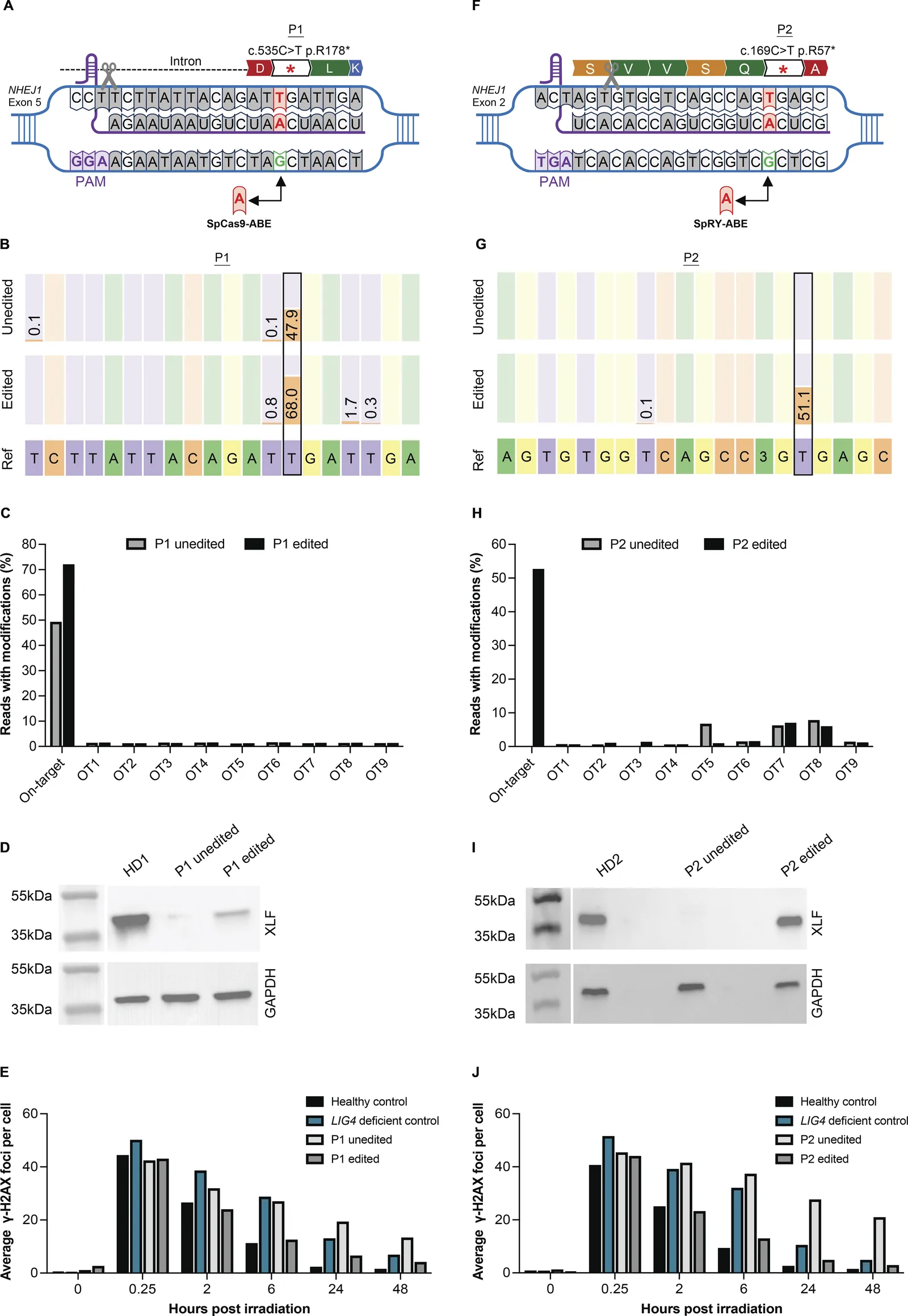
**Base editing reversal to confirm the pathogenicity of** ***NHEJ1*** **sequence variants identified in two patients with RS-SCID.** **(A)** A bespoke sgRNA was designed to reverse a compound heterozygous mutation (P1) c.532C>T p.R178* (red) with SpCas9-ABE using a canonical distal 5′-NGG-3′ PAM (purple). The variant was restored through A>G conversion (green) of the target nucleotide A7 on the non-coding strand with the PAM located at positions 21–23. **(B)** A>G conversions detected across the protospacer 7 days after electroporation of SpCas9-ABE mRNA and sgRNA to P1 fibroblasts. Reference and NGS sequences of the non-coding are shown with A7 marked as the target nucleotide. Editing was performed and confirmed by Sanger sequencing in three experiments, with one undergoing additional NGS (GENEWIZ) and bioinformatic analysis (CRISPResso2). **(C)** A>G conversions were investigated for nine possible “off-target” (OT) sites predicted by Cas-OFFinder, in unedited and edited P1 fibroblasts. Genomic DNA was isolated and amplicons generated by PCR for each site. These were then assessed by NGS (GENEWIZ) and bioinformatic analysis (CRISPResso2), with <5% above controls. **(D)** XLF (33 kDa) expression with densitometric assessment of western blot (ImageJ) with whole-cell lysate from unedited and base edited P1 fibroblasts, with a healthy donor (HD1) control. XLF levels were normalized to a GAPDH housekeeping control (36 kDa) and calculated relative to healthy donor levels. Representative data from three experiments. **(E)** Quantification of DSB repairs over a 48 h period following reversal. P1 unedited and edited fibroblasts, as well as a healthy donor control and a ligase 4 (*LIG4*)-deficient control were exposed to 3 Gy ionizing radiation and cultured. Cells were fixed, permeabilized and stained with 4′,6-diamidino-2-phenylindole dihydrochloride (DAPI) and γ-H2AX antibody, with γ-H2AX foci counted in at least 300 cells in two experiments. **(F)** A sgRNA was designed to reverse a homozygous mutation (P2) c.169C>T p.R57* (red) in combination with SpRY-ABE utilizing a distal 5′-NRN-3′ PAM (purple). The mutation was restored through A>G conversion (green) of the target nucleotide A5 on the non-coding strand with the PAM located at positions 21–23. **(G)** A>G conversions across the protospacer 14 days after electroporation with SpRY-ABE mRNA and sgRNA to P2 fibroblasts with the A5 target position marked. Amplicons covering the target nucleotide and PAM were generated and Sanger sequenced in two experiments with one undergoing additional NGS assessments. **(H)** A>G conversions were investigated for nine predicted “off-target” (OT) sites in unedited and edited P1 fibroblasts using NGS (GENEWIZ) and CRISPResso2, with <5% above controls. **(I)** Western blot with densitometric analysis of XLF expression (33 kDa) in whole-cell lysate extracted from unedited and edited P2 fibroblasts, normalized to GAPDH (36 kDa) and calculated relative to a healthy donor (HD2) control, and shown in a representative image from two experiments. **(J)** Rescue of DSB repair for P2 was confirmed after base editing of fibroblasts, with healthy donor and ligase 4 (*LIG4*)-deficient controls, by quantification of γ-H2AX foci in two experiments. Source data are available for this figure: SourceData F1.

P1 exhibited early growth retardation as well as microcephaly, and recurrent viral infections subsequently prompted immunological investigations at 16 mo of age revealing hypogammaglobulinemia (IgG 1.6 g/L, IgM 1.06 g/L, and IgA 0.45 g/L), reduced T (430/μl) and B (30/μl) cells with minimal TRECs (173/10^6^) and abnormal, non-Gaussian TCR Vβ spectratyping. Whole genome sequencing (WGS) and trio-variant analysis detected a paternal 1.8 kb microdeletion of exon 2 and a maternal sequence variant in exon 5 (c.532C>T p.R178*) of *NHEJ1* (ClinVar ID 983) (3). Protein expression was absent on Western blot and RS testing of P1 fibroblasts confirmed impaired DSB repair consistent with RS-SCID. Mutation reversal of c.532C>T was achieved in proliferating fibroblasts by precise noncoding strand A>G editing (at position A7, Fig. 1 A) using a canonical ABE incorporating a SpCas9 nickase, which was electroporated as mRNA in combination with a sgRNA using a 5′-NGG-3′ protospacer adjacent motif (PAM) (Fig. 1 A). After 7 days, next-generation sequencing (NGS) detected 20% A>G conversion at position A7 (Fig. 1 B), with minimal editing activity of bystander nucleotides or off-target editing at nine *in silico* predicted sites (Fig. 1 C). Western blot confirmed the restoration of XLF protein to 13% of healthy donor control levels (Fig. 1 D). The ability of cells to repair IR-induced DSBs was reassessed by irradiating fibroblasts at 3 Gy and counting γ-H2AX foci, which accumulate at the sites of unrepaired DSBs, over a 48-h period. DSB repair was rescued to healthy donor levels following base editing, while original P1 fibroblasts and an impaired ligase IV–mutated control remained sensitive (Fig. 1 E). These findings confirmed the pathogenicity of c.532C>T and established the feasibility of mutation reversal by base editing in patient fibroblasts.

## P2: *NHEJ1* c.169C>T p.R57* reversal with SpRY-ABE

P2 exhibited microcephaly and immunological investigations at 5 years of age revealed very low T (80/μl), B (50/μl) and NK (60/μl) cell counts, and hypogammaglobulinemia with low IgG (1.51 g/l) and raised IgM (7.1 g/l). Thymic output was reduced with negligible TRECs (38 × 10^6^), while TCR Vβ repertoire displayed a non-Gaussian distribution. Whole exome sequencing (WES) revealed a homozygous variant c.169C>T p.R57* (Clinvar ID 3336660) in exon 2 of *NHEJ1* (Fig. 1 F) (4). A sgRNA was designed to reverse the variant by installing an A>G conversions on the non-coding strand, while a relaxed PAM variant ABE (SpRY-ABE) compatible with sgRNA using a 5′-NRN-3′ PAM was electroporated as mRNA into proliferating fibroblasts (5). NGS analysis after 14 days revealed 50% A>G conversion at the target nucleotide A5 with no detectable bystander editing (Fig. 1 G) or off-target editing at nine *in silico* predicted sites (Fig. 1 H). XLF expression was restored to 74% of healthy donor control levels (Fig. 1 I), while DSB repair was also rescued (Fig. 1 J). These findings together confirmed pathogenicity of this *NHEJ1* sequence variant through targeted ABE-mediated gene correction.

## Discussion

Investigation of sequence variants suspected of causing serious immune disorders can be time consuming and impracticable ahead of major interventions such as allogenic hematopoietic stem cell transplantation (allo-HSCT). In the case of suspected RS disorders, systematic targeted sequencing may uncover variants in genes of clinical relevance. Fibroblast cultures are initiated during the diagnostic process and exposure to ionizing radiation may reveal impaired DNA repair pathways (1). RS has long been an important predictive factor in allo-HSCT, and defining the underlying genetic defects provides additional categorization and risk stratification. Widespread application of high throughput WES or WGS has resulted in more patients being identified, with variable phenotypes. Sequencing may reveal likely-pathogenic mutations, or variants of unknown significance, which can then be further investigated in functional studies. The availability of genome editing technology has provided opportunities to model and reverse genetic changes, including in primary fibroblasts or derived induced pluripotent stem cells (iPSCs). Canonical CRISPR/Cas9 nucleases create precise DSBs, which can be repaired by HDR using DNA templates for sequence correction. However, this approach relies on endogenous repair pathways, which may be impaired in RS disorders. Alternatively, base editing uses deactivated Cas9 nickases to direct precise A>G (or C>T) deamination without DSB formation. We therefore reasoned this could offer a suitable platform to reverse pathogenic mutations in patients with RS disorders. Proof of concept investigations were undertaken for two *NHEJ1* mutations uncovered during recent referrals to our tertiary immunodeficiency service. Primary fibroblasts had been previously isolated for clinical diagnostic RS testing after suspected mutations had been identified in both *NHEJ1* kindreds. We were able to reverse the first using appropriate sgRNAs and standard SpCas9-ABE editor, while the second required flexible PAM recognition afforded by SpRY-ABE (2, 5). In both cases, mRNA stocks for each editor were pre-available and already validated, while sgRNAs could be rapidly designed and procured from commercial suppliers. Electroporation conditions and assessment of editing of primary fibroblasts were in place, including controls for editing of HLA-class I expression through *B2M* editing. Sequencing verified genetic correction, and although off-target screens applied were largely bioinformatic, on-target correction was supported by blotting of protein expression and testing for RS. Given that primary fibroblast isolation, culture and expansion requires around 6 wk, we anticipate it will be feasible to incorporate editing investigations into a first-pass strategy for variants of unknown significance, depending if additional investigations are required due to inefficient or aberrant editing. This strategy should be suitable for other defects of DNA repair reliant on similar assays such as ligase IV and Artemis deficiencies, with around 70% of existing mutations in ClinVar databases amenable to similar corrections. Furthermore, related tools including prime editing offer a possibility to investigate additional changes not amenable to base editing, including transversions, insertions, or deletions. Genome editing platforms continue to evolve and are starting to be applied therapeutically for disease correction in HSCs to support autologous alternatives to allo-HSCT. The ability to rapidly assess editing reagents in fibroblasts, or other somatic cells such as T or B cells when available, should provide critical information regarding the feasibility and fidelity of selected editing reagents to correct autologous HSCs for therapeutic purposes. Additional validatory studies may involve *in vitro* differentiation to assess V(D)J recombination and resulting T and B cell receptor repertoire development, as well as models of human HSC engraftment and differentiation in immunodeficient mice. Our findings suggest that base editing and other similar approaches can be readily adopted to aid characterization and investigation of sequence variants as part of existing diagnostic pathways. Such investigations will also play a critical role in supporting the accelerated deployment of patient-specific gene correction strategies as alternative therapeutic interventions are considered.

## Ethics statement

Fibroblasts were obtained with informed consent from patients and their parents (IRAS project ID 325015) under Research and Ethics Committee approval (23/WM/0249).

## Supplementary Material

SourceData F1 is the source file for Fig. 1.

## Data Availability

Data are available from the corresponding author upon reasonable request.
