# Supplementary material for: Base editing reversal of radiation sensitivity in NHEJ1 immunodeficiency
Source: J Hum Immun. 2026 Sep 3;2(6):e20260068. doi: 10.70962/jhi.20260068 (PMC13540376; doi:10.70962/jhi.20260068)
Supplement: Supplementary file 1 [file jhi_20260068_sourcedataf1.pdf]

Fig 1D

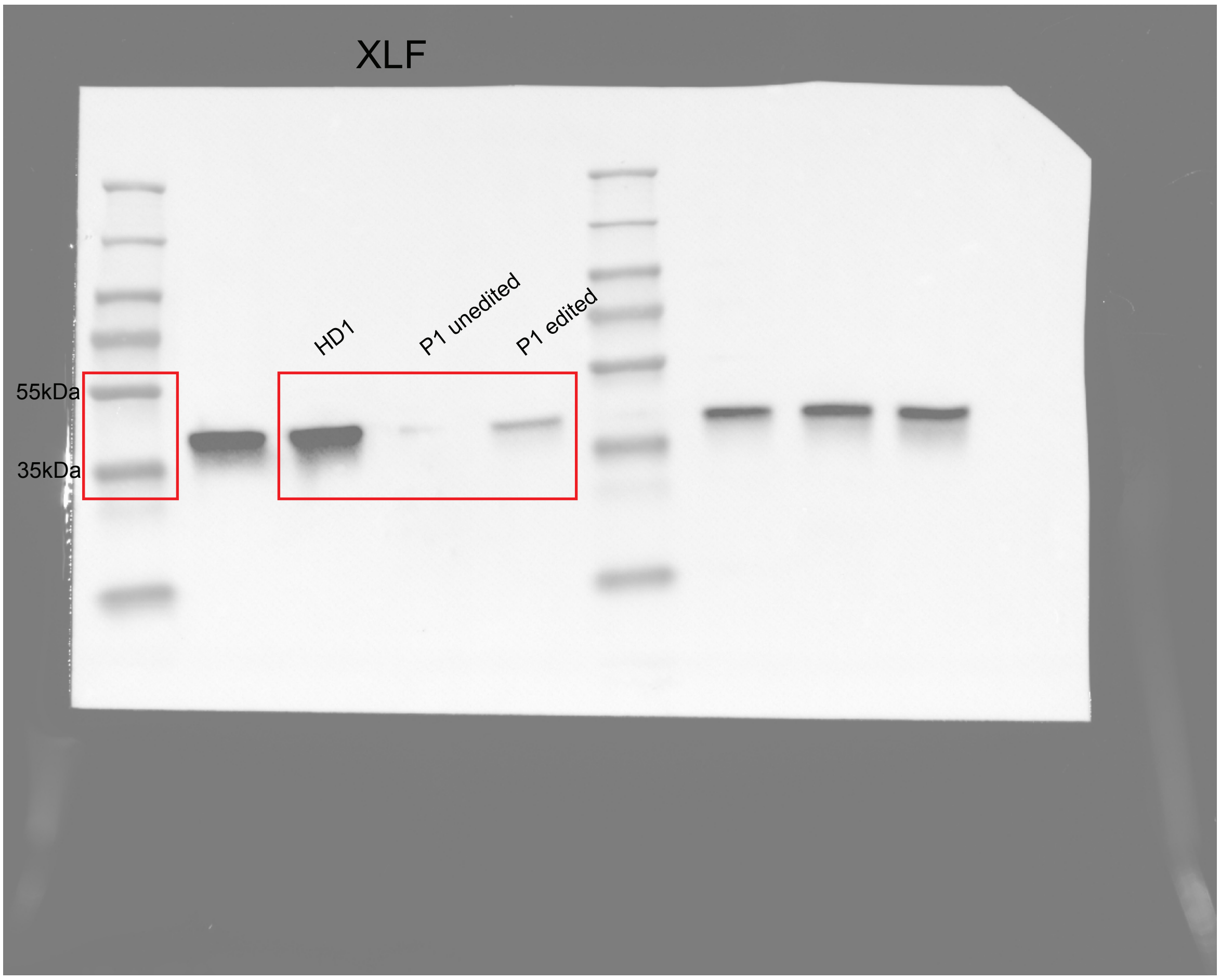
XLF
HD1
P1 unedited
P1 edited
55kDa
35kDa

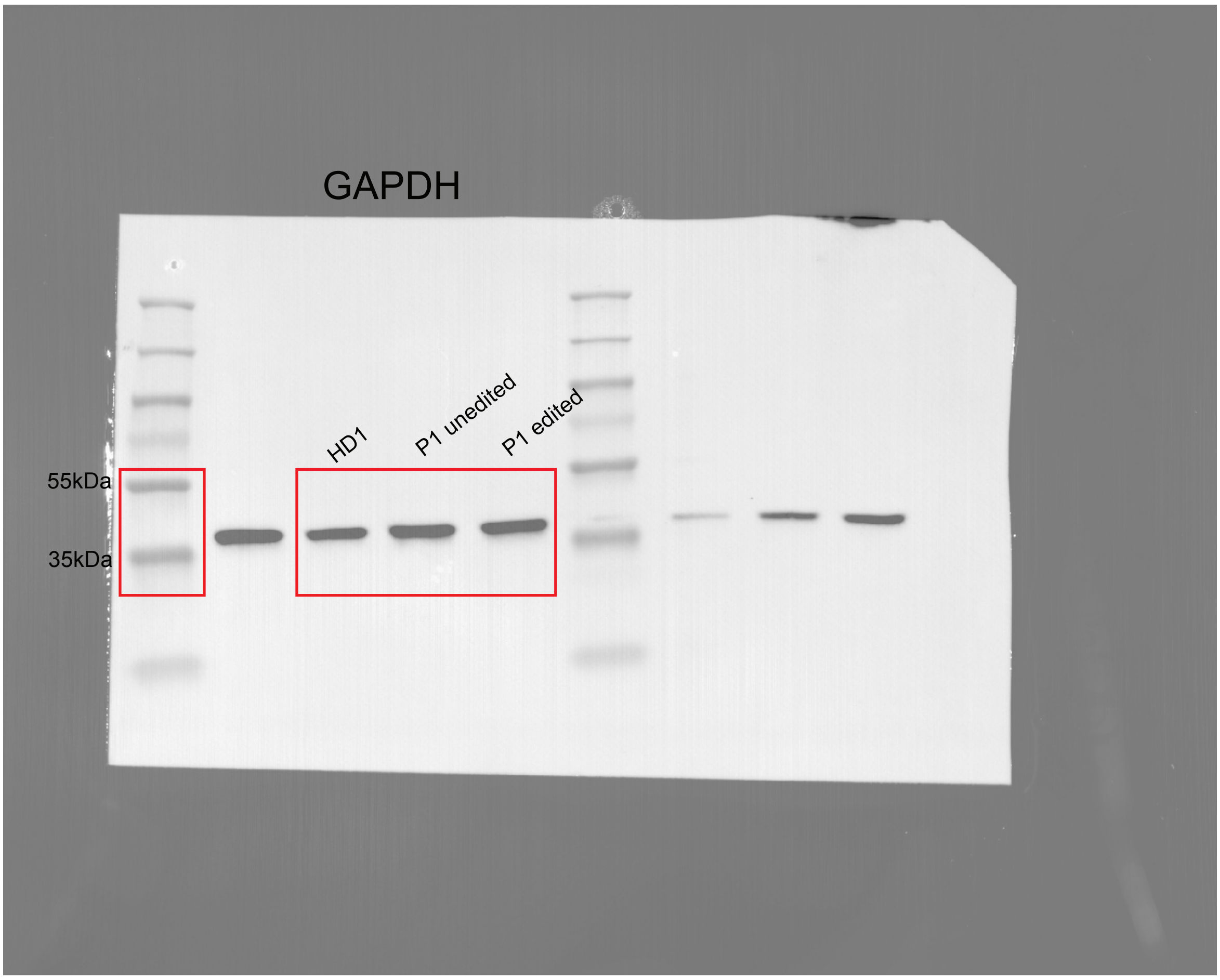
GAPDH
HD1
P1 unedited
P1 edited
55kDa
35kDa

Fig 1I

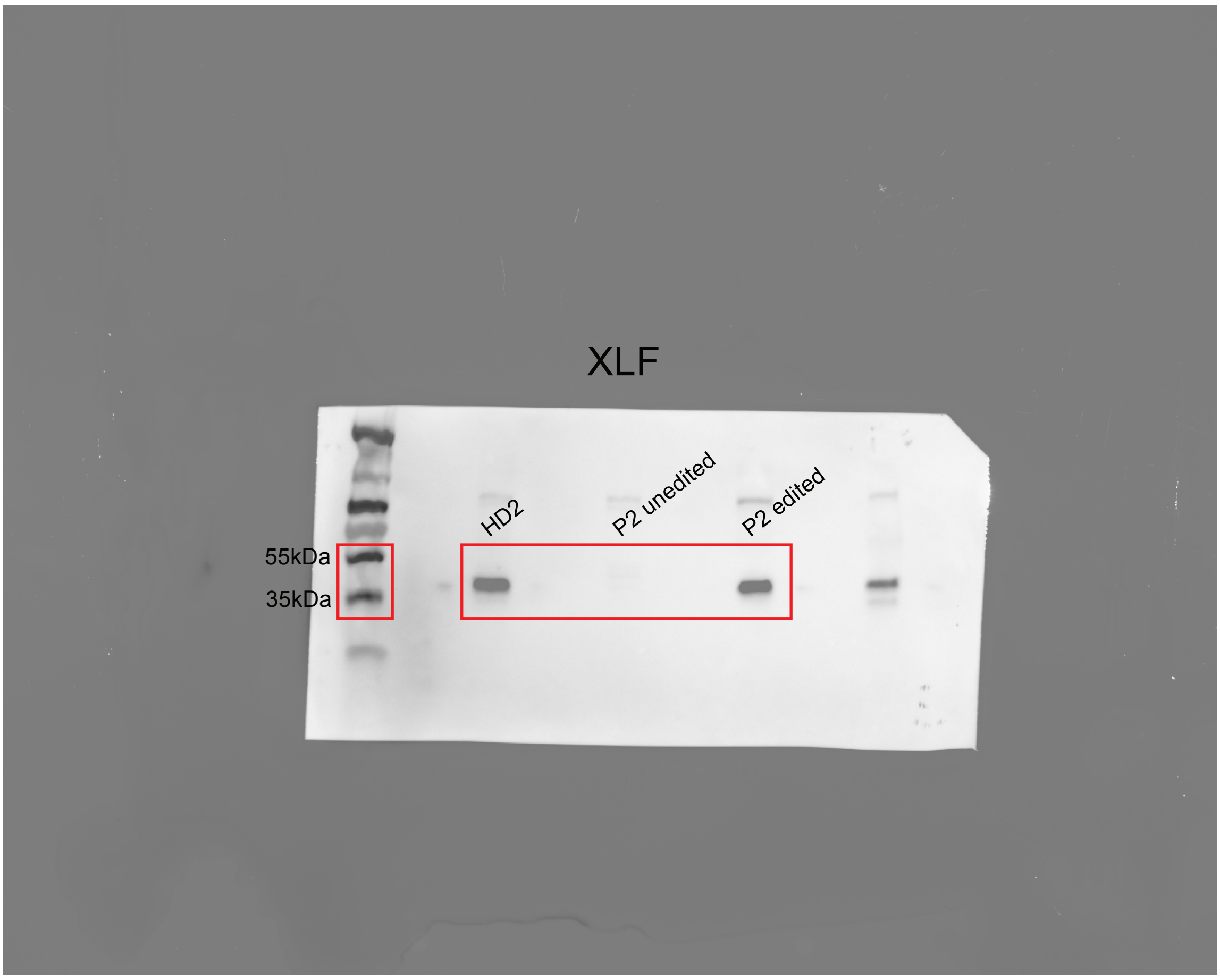
XLF
HD2
P2 unedited
P2 edited
55kDa
35kDa

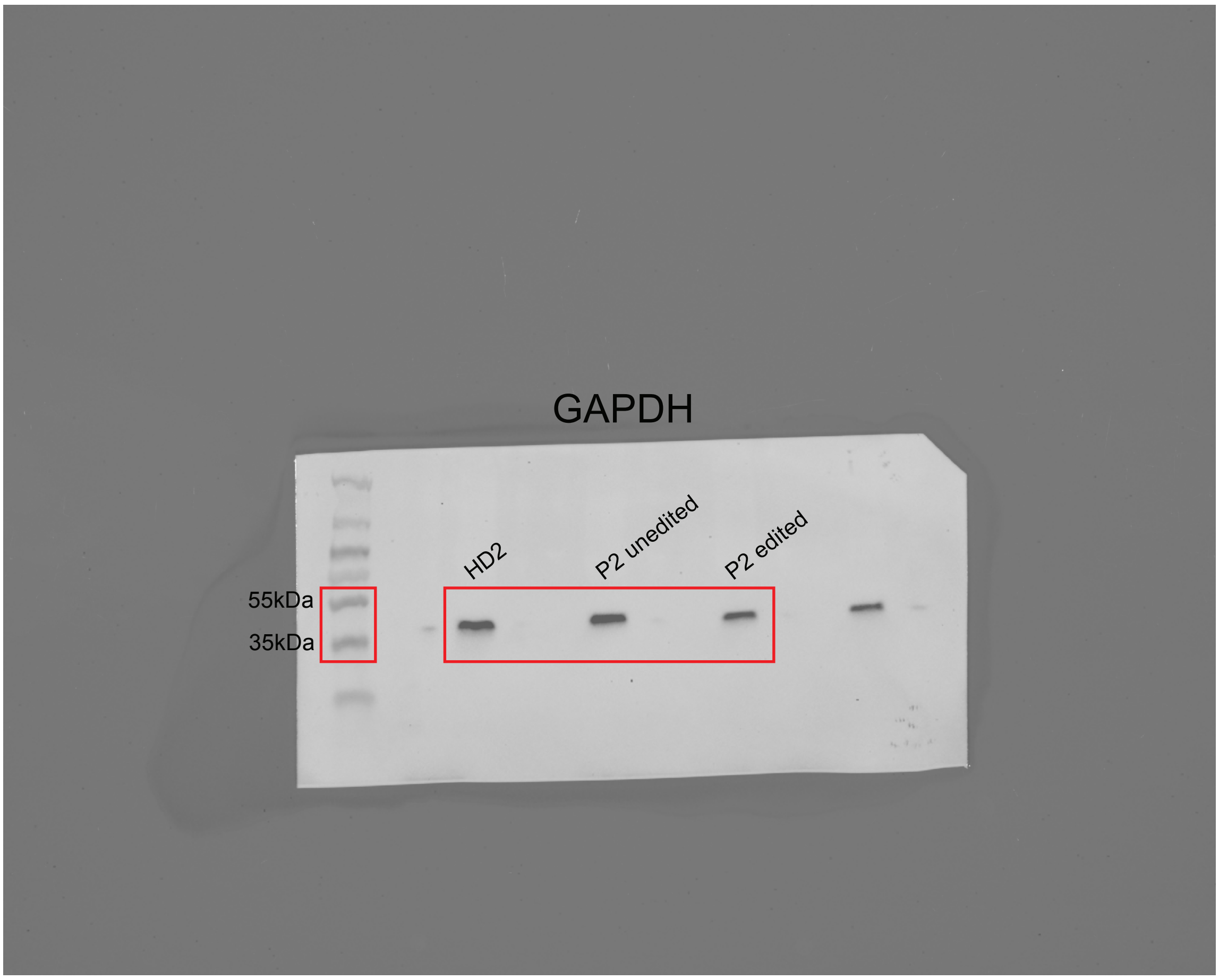
GAPDH
HD2
P2 unedited
P2 edited
55kDa
35kDa
